# Compositional shifts in root-associated bacterial and archaeal microbiota track the plant life cycle in field-grown rice

**DOI:** 10.1371/journal.pbio.2003862

**Published:** 2018-02-23

**Authors:** Joseph A. Edwards, Christian M. Santos-Medellín, Zachary S. Liechty, Bao Nguyen, Eugene Lurie, Shane Eason, Gregory Phillips, Venkatesan Sundaresan

**Affiliations:** 1 Department of Plant Biology, University of California-Davis, Davis, California, United States of America; 2 Department of Agriculture, Arkansas State University, Jonesboro, Arkansas, United States of America; 3 Department of Plant Sciences, University of California-Davis, Davis, California, United States of America; Massachusetts Institute of Technology, United States of America

## Abstract

Bacterial communities associated with roots impact the health and nutrition of the host plant. The dynamics of these microbial assemblies over the plant life cycle are, however, not well understood. Here, we use dense temporal sampling of 1,510 samples from root spatial compartments to characterize the bacterial and archaeal components of the root-associated microbiota of field grown rice (*Oryza sativa*) over the course of 3 consecutive growing seasons, as well as 2 sites in diverse geographic regions. The root microbiota was found to be highly dynamic during the vegetative phase of plant growth and then stabilized compositionally for the remainder of the life cycle. Bacterial and archaeal taxa conserved between field sites were defined as predictive features of rice plant age by modeling using a random forest approach. The age-prediction models revealed that drought-stressed plants have developmentally immature microbiota compared to unstressed plants. Further, by using genotypes with varying developmental rates, we show that shifts in the microbiome are correlated with rates of developmental transitions rather than age alone, such that different microbiota compositions reflect juvenile and adult life stages. These results suggest a model for successional dynamics of the root-associated microbiota over the plant life cycle.

## Author summary

Plant roots are colonized by complex communities of bacterial and archaeal microbiota from the soil, with the potential to affect plant nutrition and fitness. Although root-associated microbes are known to have the potential to be utilized to promote crop productivity, their exploitation has been hindered by a lack of understanding of the compositional dynamics of these communities. Here we investigate temporal changes in the root-associated bacterial and archaeal communities throughout the plant life cycle in field-grown rice over multiple seasons and locations. Our results indicate that root microbiota composition varies with both chronological age and the developmental stage of the plants. We find that a major compositional shift correlates with the transition to reproductive growth, suggestive of distinct root microbiota associations for the juvenile and adult plant phases. The results from this study highlight dynamic relationships between plant growth and associated microbiota that should be considered in strategies for the successful manipulation of microbial communities to enhance crop performance.

## Introduction

Plants assemble soil-derived root-associated microbial communities referred to as microbiota [1–3]. Taxa within the root-associated microbiota have been found to be beneficial for plant growth and resistance to biotic and abiotic stresses [4,5]. Previous studies on plant microbiota have primarily characterized the bacterial and archaeal communities, and in what follows, the term microbiota encompasses these communities. Plants host root-associated microbiota in 3 spatially distinct root compartments with significantly different compositions: the soil adjacent to the root (the rhizosphere), the root surface (the rhizoplane), and the root interior (the endosphere) [6–8]. Each root-associated compartment has significantly different compositional profiles from the communities in unplanted soil, thus indicating the roots enrich for subsets of the soil microbiota [6–10]. It has been suggested that enrichment of copiotrophic microbes occurs in the rhizosphere, whereas unplanted soils are enriched for oligotrophic microbes [11]. This enrichment process and how the root-associated microbiota change throughout the life cycle of the plant remain poorly characterized.

Limited information is available about the spatiotemporal dynamics of the plant root-associated microbiota, especially for the root endosphere. Previously, we characterized temporal progressions of the microbiota across the rhizosphere-endosphere continuum of greenhouse grown rice seedlings from transplantation to 2 weeks after transplantation [8]. Over this relatively short period of time, the microbiota shifted to become more similar to those hosted by rice plants that were 6 weeks old, suggesting that the endospheric microbiota reaches a steady state. From that study, however, we were unable to formulate how root microbiota change over the full life cycle of rice plants.

The most systematic study available on the possible impact of plant developmental stage on the composition of the plant root microbiota has been on the perennial plant *Arabis alpina* [12]. Analysis of the rhizosphere and root microbiota during 3 time points encompassing a 28-week period under greenhouse conditions showed changes in the microbiota with soil residence time [12]. An early flowering mutant had no significant effect on microbiota compared to the nonflowering wild-type plants, suggesting that the observed changes were due to soil residence time, rather than plant development stage. These data supported a model in which “after microbiota acquisition during vegetative growth, the established root-associated bacterial assemblage is structurally robust to perturbations caused by flowering and drastic changes in plant stature” [12]. However, it is unknown how root-associated microbiota might vary on weekly timescales from seedling to maturity under field conditions for annual plants, what patterns of change can be expected over the annual life cycle, and whether microbiota assemble in a consistent manner across geographically and climatically distinct regions.

Given the likely role that root-associated microbiota play in conferring beneficial properties to the host plants [1], it is important to understand how root microbiota are structured over the life cycle of their crop hosts and whether temporal shifts are consistent between geographic locations. Rice is a staple crop for a large proportion of the world population [13], and rice cultivation is a major contributor to greenhouse gas emissions arising from microbial activity [14]. Therefore, in this study, we use a high-resolution spatiotemporal approach to detail the successional progression of the microbiota across the rhizosphere, rhizoplane, and endosphere compartments over the life cycle of rice plants grown under field conditions over 3 consecutive growing seasons as well as field sites from 2 geographically distinct regions of the United States. Using related rice varieties with different developmental rates grown under field conditions, we conclude that in contrast to predictions of prevailing models, shifts in the root microbiome are correlated with age and developmental stages and that different microbiota compositions reflect juvenile and adult life stages. Partial validation of these hypotheses was obtained by successfully utilizing computational models to predict plant age.

## Results

### Microbiomes are shaped by distance from the root, growing regions, and plant age

**Fig 1:**
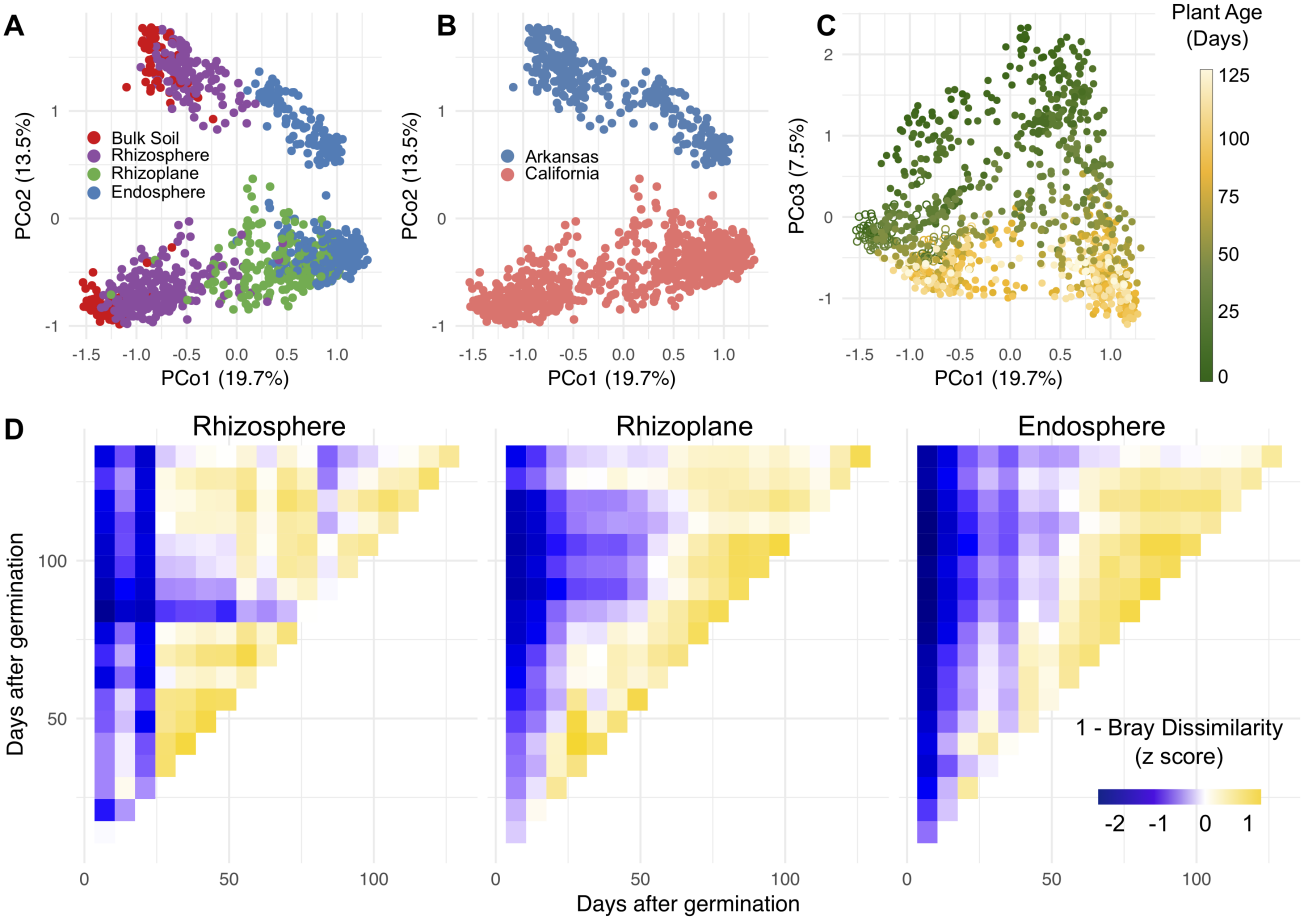
The root-associated microbiota stabilizes after 8–9 weeks after germination. (A) Principal coordinates analysis (PCoA) of Bray-Curtis dissimilarity between samples colored by root compartment. The numerical values used to construct this figure can be found in S1 Data. (B) The same plot as in panel A but colored by the field location. (C) The same analysis as in panels A and B but now showing principal coordinate (PCo) 1 versus PCo3, and the points are colored by the age of the plants from which the samples were taken. Hollow points represent bulk soil samples. (D) Heatmaps showing mean pairwise z-scores for similarity, computed as (1 − Bray-Curtis dissimilarity), between time points in each compartment for the 2014 California samples.

To investigate the bacterial and archaeal microbiota associated with the rhizosphere, rhizoplane, and endosphere of rice roots as well as those associated with unplanted soil over the growing season, we sequenced the V4 region of the 16S rRNA gene of 1,510 samples, revealing 10,893 different operational taxonomic units (OTUs). Depending on the season, we collected plants from the field sites either weekly or every other week (see Materials and methods). To visualize the underlying driving forces of microbial community variation in our data, we used principal coordinates analysis (PCoA) in combination with permutational multivariate analysis of variance (PERMANOVA) on Bray-Curtis dissimilarities between the samples (S1 Table). Because of the relatively large number of samples in this dataset and the use of multiple sequencing runs, we first inspected how much variance was partitioned to sequencing run. We found that samples significantly differed by sequencing run, but this effect was minor compared to the experimental variables tested (R2 = 0.009, *P* = 0.001). We found that samples from 2014 and 2015 California samples as well as the 2016 Arkansas samples display a spatial pattern of divergence along the first principal coordinate, where communities in bulk soil samples cluster on one end of the axis and endosphere communities cluster on the other (Fig 1A). PERMANOVA of pairwise distances between microbial communities indicated that the microbiota differed significantly between root-associated compartments (R2 = 0.22, *P* < 0.001), in agreement with prior observations [6–9,11,15]. The microbiome samples from Arkansas and California separated along the second principal coordinate (Fig 1B). This observation was supported by the PERMANOVA statistic (R2 = 0.13, *P* < 0.001). The soil from the Arkansas and California field sites differs in that the Arkansas site has a sandy loam soil compared to the California site, which has a silty clay soil (https://websoilsurvey.sc.egov.usda.gov). Chemical analysis showed that the California soil had higher concentrations of potassium, sodium, calcium, magnesium, organic matter, and pH content than the Arkansas soil (S2 Table). Arkansas soil had greater concentrations of nitrate and phosphorus than the California site.

We next measured the effect of plant age on the root-associated microbiota. We found that this effect is observable across the third principal coordinate of the PCoA plot and is consistent across the 2 growing regions and across the 2 growing seasons within California (Fig 1C). The amount of variance partitioned to the effect of plant age on the root-associated microbiota was comparable between the 2 growing regions (California: R2 = 0.093, *P* < 0.001; Arkansas: R2 = 0.080, *P* < 0.001). We observed that the rhizoplane and endosphere microbial communities shift over the first 7 to 8 weeks after germination but stabilize thereafter (Fig 1D, S1 Fig). This pattern was observed over both growing seasons in California and also in the Arkansas field trial. However, a stabilization pattern was not observed in the rhizosphere microbial communities (Fig 1D, S1 Fig) despite the rhizosphere microbiota showing distinct shifts over the season (Fig 1C). It is interesting to note that the selected rice varieties grown in California and Arkansas reach panicle initiation (entry into reproductive growth) 8–9 weeks after germination in their respective locations, suggesting a correlation between plant developmental stage and root-associated microbiota dynamics.

The multiyear sampling scheme of our experimental design allowed us to quantify the effect of year-to-year variation on the root-associated microbiota. Although the root-associated microbiota varied significantly across the 2 different years at the California site, the effect was relatively small (R2 = 0.01, *P* = 0.001) compared to the other factors analyzed within this experiment. Together, these data suggest that the root-associated microbiota shifts in each root-associated compartment during the vegetative growth stage of the season and stabilizes upon entry into reproduction and that these patterns are consistent across different growing seasons.

### Microbiota dynamics over the season are marked by increasing and decreasing shifts in relative abundance of specific phyla

**Fig 2:**
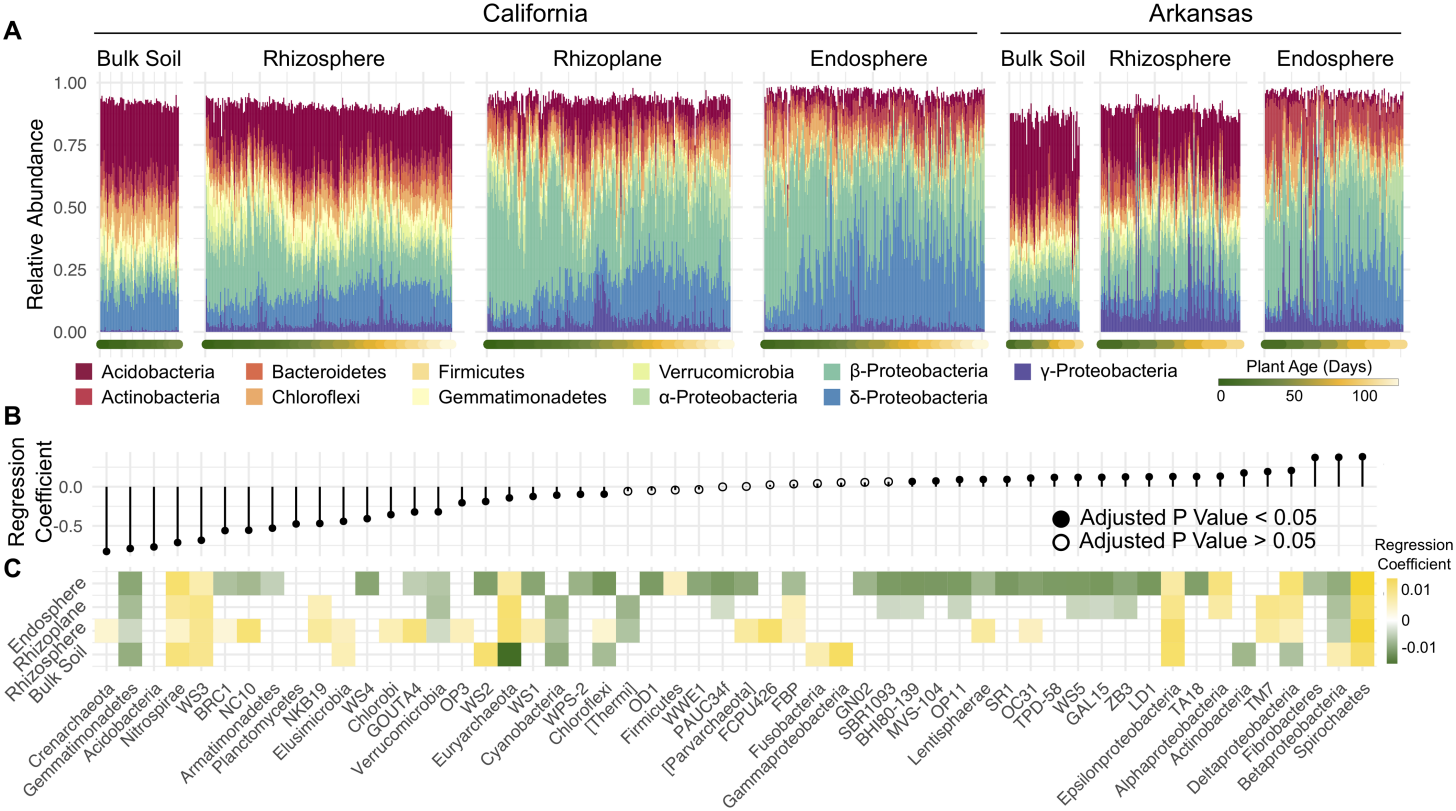
Shifts in the microbiota over time are associated with increasing and decreasing phyla. (A) Bar plots of the top 11 phyla abundances over the course of the seasons in each compartment. Each bar represents 1 sample that was taken throughout the course of the growing season. The bars are ordered by the age of the plant as indicated by the colored points beneath each bar. Both the 2014 and 2015 data were used for this graph. The numerical values used to construct panel A can be found in S2 Data. (B) Beta regression coefficient estimates for microbial phyla that are either increasing (above 0) or decreasing (below 0) in relative abundance from the outside of the root to the inside of the root. (C) Beta regression coefficient estimates for microbial phyla that are increasing (above 0) or decreasing (below 0) in relative abundance over the course of the seasons in each compartment. All regression coefficients used to construct panels B and C can be found in S3 Data.

We next sought to characterize the specific phyla responsible for the significant differences between the root-associated compartments and how these various phyla change in abundance over the course of the season. Overall, we noticed similarities in patterns in phyla abundance over time between the 2 growing regions (Fig 2A). Because the *Proteobacteria* phylum contains a broad phylogenetic makeup and because *Proteobacteria* make up a large proportion of the rice root-associated microbiota, we further divided the *Proteobacteria* phylum into its respective classes for this analysis. To model increasing or decreasing relative abundance of individual phyla between the various root-associated compartments, we assigned each compartment a value relative to its spatial position: bulk soil was position 0, rhizosphere was position 1, rhizoplane was position 2, and endosphere was position 3. We modelled how each phylum either increased or decreased over these positions using beta regression. Beta regression is used for modeling dependent variables that lie in the interval (0, 1) and is thus useful for modelling individual taxa as a relative proportion of the total microbial community.

Using this method, of the 55 detectable phyla, we identified 42 phyla that significantly differed in spatial distribution from the exterior to the interior of the root (Fig 2B). There were 20 phyla whose relative abundance significantly increased from the soil environment to the root interior and 22 microbial phyla whose relative abundance decreased from the soil environment to the root interior. The absolute values of the regression coefficients were overall greater for root-depleted phyla, suggesting that a smaller proportion of the soil microbiota is enriched by the plant, while more microbial taxa are depleted.

We again performed beta-regression within each compartment to identify phyla that significantly increased or decreased in relative abundance as a function of plant age (Fig 2C). In general, trends in abundance over the season were consistent across the rhizocompartments (excluding the bulk soil) for each phylum. For instance, in the rhizosphere, rhizoplane, and endosphere, *Betaproteobacteria*, *Verrucomicrobia*, and *Gemmatimonadetes* consistently decreased over the course of the season, while *Nitrospirae*, *WS3*, *Deltaproteobacteria*, *Epsilonproteobacteria*, *Euryarchaeota*, and *Spirochaetes* all increased. In the rhizoplane and endosphere, most of the taxa with dynamic abundance patterns significantly decreased over the course of the season (12/22 and 32/40, respectively), suggesting that the root-associated compartments are initially colonized by a diverse set of microbes from the soil but are either eventually outcompeted by other taxa or are selected against by the host plant. Together, these results indicate that despite large differences in microbiota composition between the growing regions, consistent trends in abundance at the phylum level define microbiota differences between the rhizocompartments and over the course of the growing season.

### Plant age is predicted by subsets of the microbiota that are conserved across field sites

**Fig 3:**
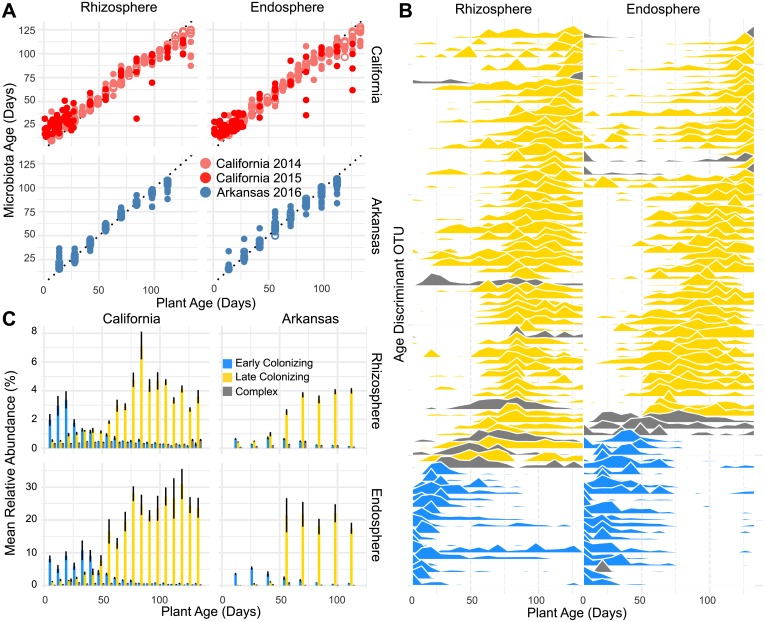
Random forest model detects taxa that are accurately predictive of plant age. (A) The result of predicting plant age using the sparse random forest (RF) models for the 2014 and 2015 season. Each point represents a predicted age value. Solid circles represent predictions from training data, while hollow points represent predictions from test data. The numerical values used to construct panel A can be found in S4 Data. (B) Abundance profiles for the age-discriminant operational taxonomic units (OTUs) in rhizosphere and endosphere compartments over the course of the California 2014 growing season. OTUs are ordered along the *y*-axis by timing of peak abundance. The orders of the OTUs on the *y*-axis are not shared between rhizosphere and endosphere despite both the models for each compartment sharing a subset of age-discriminant taxa. OTUs are colored by their classification as early, late, or complex colonizers over the season (see color scale in panel C). See S3 Table for order of OTUs. There were 18 OTUs with decreasing patterns, 7 OTUs with complex dynamic patters, and 60 OTUs with increasing dynamic relative abundances in the rhizosphere. In the endosphere, there were 22 OTUs with decreasing relative abundances, 7 OTUs with complex patterns, and 56 with increasing relative abundances over the season. Slope estimates used to classify OTUs as having early, late, or complex patterns can be found in S5 Data. (C) Mean total abundance for the age-discriminant taxa across sites and compartments. The numerical values used to construct panel C can be found in S7 Data.

We next sought to identify individual microbial OTUs that could be used to discriminate plant age. We identified 85 of the most important age-discriminant OTUs from the rhizosphere and endosphere from full random forest (RF) models and used these OTUs to develop a more accurate sparse RF model for each compartment to predict plant age (see Materials and methods). We found the sparse RF models explained 91.5% and 88.4% of the variance related to plant age for the rhizosphere and endosphere, respectively. Plant age was accurately predicted by the compartment-specific sparse RF models for the California and Arkansas sites (Fig 3A). Additionally, we note that the ages of plants sampled from California during the 2015 season were able to be accurately predicted, despite the models not being trained on this data. These results indicate that specific sets of microbes in both the endosphere and rhizosphere behave in consistent patterns over the course of the rice plant’s life cycle between seasons and across geographic regions.

We next examined the phylogenetic composition of the 85 OTUs used for plant age prediction in the rhizosphere and endosphere sparse models. The OTUs used in the sparse RF models were phylogenetically diverse. In the endosphere model, the model used OTUs belonging to 10 phyla, 19 classes, 29 orders, and 37 families, while the rhizosphere model used OTUs belonging to 11 phyla, 24 classes, 37 orders, and 45 families. Consistent with general trends in alpha diversity between the compartments (S2 Fig), the rhizosphere model contained a more phylogenetically diverse set of microbes for classification. We used a linear model approach to classify the OTUs included in each model as being early (significantly decreasing slope over the course of the season), late (significantly increasing slope over the course of the season), or complex (slope not significantly different than 0 over the course of the season) colonizers over the course of the season (Fig 3B). We found that for both the rhizosphere and endosphere sparse RF models, most of the OTUs were late colonizers, while fewer OTUs were classified as complex or early colonizers (Fig 3B). The rhizosphere and endosphere models shared 22 OTUs, 20 of which were in agreement for early/late/complex colonizer classification. Within the OTUs classified as late or early colonizers for the rhizosphere and endosphere models, OTUs belonging to the *Betaproteobacteria* class were the most represented class (Fig 3B). At the order level, however, the OTUs making up the increasing or decreasing fraction within the sparse models were significantly different (S3 Fig). *Betaproteobacteria* OTUs classified as early colonizers were mainly composed of *Burkholderiales*, while the late colonizing OTUs were mainly composed of *Rhodocyclales* and *SBla14*. We also noticed differences between the rhizosphere and endosphere RF models for the taxa they were using for classification. The endosphere model used many more *Alphaproteobacteria* OTUs than the rhizosphere model, which mirrors differences in trends at the phylum level (Fig 2). Similarly, the rhizosphere model used OTUs from the phylum *Verrucomicrobia*, whose relative abundance at the phylum level is significantly higher in the bulk soil and rhizosphere than the endosphere (Fig 2B).

We next evaluated the fractional contribution of the age-discriminant OTUs to the total microbiota over the course of the season (Fig 3C). Overall, the age-discriminant OTUs composed a greater proportion of reads in the endosphere compared to the rhizosphere. This was consistent across both the California and Arkansas sites. As expected, the early colonizer OTUs were dominant at the beginning of the season, while those identified as increasing in relative abundance were dominant at the end of the season. Interestingly, we noticed that the switch in dominance between early/late colonizing classified OTUs occurred 8 to 9 weeks after germination, coinciding with the switch from vegetative to reproductive growth for the included varieties.

We next asked if the early colonizing age-discriminant OTUs were significantly enriched in their respective root compartments compared to unplanted soil controls. We were only able to use the early time points (less than or equal to 49 days after germination) as a comparison because of the inability to gather bulk soil controls in the latter part of the season. We found that the early colonizing microbes in the RF models were predominantly enriched in the rhizosphere and endosphere compared to bulk soil controls (S4 Fig). The late colonizing microbes had more similar relative abundance patterns to the bulk soil control samples. Because this analysis was constrained to using the earlier time points, we expect that the late colonizing OTUs would be predominantly enriched compared to the bulk soil controls in the latter time points. These results suggest that the early colonizing OTUs included in both the rhizosphere and endosphere sparse RF models are selected either actively or passively by the plant roots and their abundance profiles are likely not a product of edaphic processes separate from the plant.

### Drought stress is associated with an immature endosphere microbiota

Drought is one of the most common and devastating stresses to affect rice production around the world [16]. The activities of soil microorganisms are altered by water status [17,18] and have been implicated in alleviating drought symptoms in various plant species under laboratory conditions [19,20]. It was unknown until recently how water deprivation affects the rice root-associated microbiota [21]. Drought stress most strongly alters the endosphere microbiota (relative to the rhizosphere and bulk soil), with *Actinobacteria* and *Chloroflexi* strongly increasing in abundance and *Deltaproteobacteria* and *Acidobacteria* strongly decreasing under drought conditions. Similar patterns have been reported for the root microbiota of other grass species under drought stress [22]. Despite large changes in the rice microbiota in the face of drought stress [21], it is unknown how these changes reflect on normal development of the microbiota.

**Fig 4:**
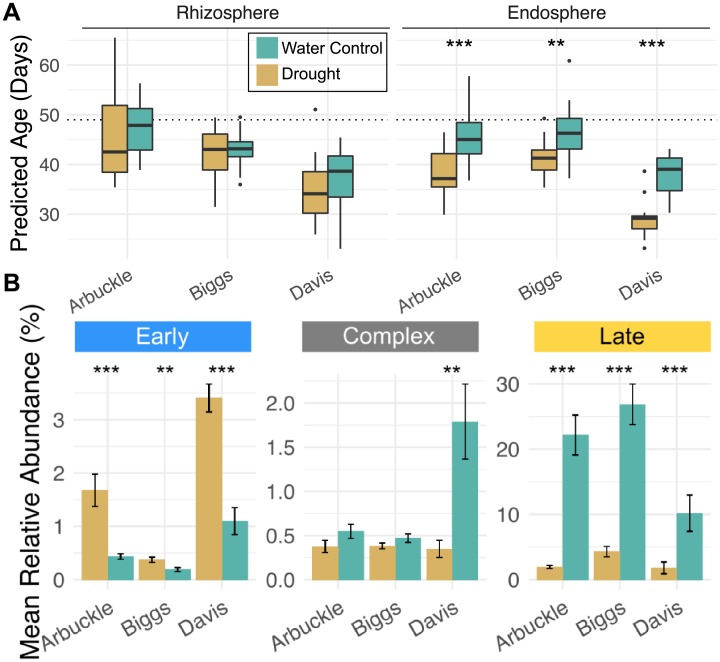
Drought exposure is associated with immature development of the endosphere microbiota. (A) Microbiota age predictions for rhizosphere and endosphere samples from well-watered and drought-exposed plants. The horizontal line represents day 49, the age at which the plants were sampled. Age predictions for the drought-treated and well-watered control microbiota samples can be found in S8 Data. (B) Abundance of age-discriminant operational taxonomic units (OTUs) in the endosphere samples of well-watered and drought-exposed plants. Color scales are shared between panels A and B. Early, late, and complex colonizers are the same 85 OTUs used in the age-predicting random forest (RF) models (Fig 3B). *** *P* < 0.001, ** *P* < 0.01, * *P* < 0.05. Numerical values used to construct panel B can be found in S10 Data.

To study how drought stress may affect normal development of the microbiota, we used our sparse age-predicting RF models in conjunction with the data collected from Santos-Medellín et al. to model plant age as a function of the microbiota (Fig 4A). Santos-Medellín et al. analyzed the rhizosphere and endosphere microbiota of 49-day-old drought-stressed and well-watered rice plants growing in 3 diverse soils under greenhouse settings [21]. When predicting ages from the samples included in this experiment, we found that the soil in which the plants were growing had the largest effect on variation in age predictions (F = 55.69, *P* < 2 × 10^−16^ ANOVA, S4 Table). The variation due to soil source was largely caused by samples originating from the Davis soil being predicted as younger compared to samples from plants grown in Arbuckle and Biggs soil. All 3 soils are classified as silty clays, and they were all comparable in chemical composition (S2 Table), but cultivation history differs between the soils: Arbuckle and Biggs soil have cultivated rice every summer season for at least the previous 8 years, while the Davis field has been fallowed for the previous 6 years [21]. Thus, it is likely that soil cultivation history can affect the accuracy of the age-predicting RF models.

Watering treatment had the second largest effect on plant age prediction (F = 32.54, *P* = 4.72 × 10^−8^ ANOVA, S4 Table). This effect was only apparent in the endosphere (adjusted *P* = 7.9 ×10^−9^, Tukey’s post hoc test, S9 Data) and not in the rhizosphere (adjusted *P* = 0.34, Tukey’s post hoc test, S9 Data). This finding is consistent with the conclusions by Santos-Medellín et al. that the endosphere microbiota is most affected by water deprivation compared to the other rhizocompartments [21]. In each soil, the drought-stressed plants hosted endosphere microbiota that were consistently less mature than well-watered plants. Because the age-discriminant OTUs are predominantly classified as late colonizers, we considered whether a fundamental shift in the endosphere microbiota could cause a decrease of all age-discriminant OTUs and therefore account for the prediction of RF models that drought-stressed rice plants host immature endosphere microbiota. While we did observe a decrease in the late colonizing age-discriminant microbes, we found that early colonizing age-discriminant microbes have significantly higher relative abundances in the drought-stressed endospheres of each soil (Fig 4B, *P* < 0.05 Tukey’s Honest Significant Difference Test, S5 Table). Together, these data indicate that drought stress is associated with an immature microbiota in the root endosphere, but not rhizosphere.

### The root-associated microbiota of distant field sites converge in similarity during the growing season

**Fig 5:**
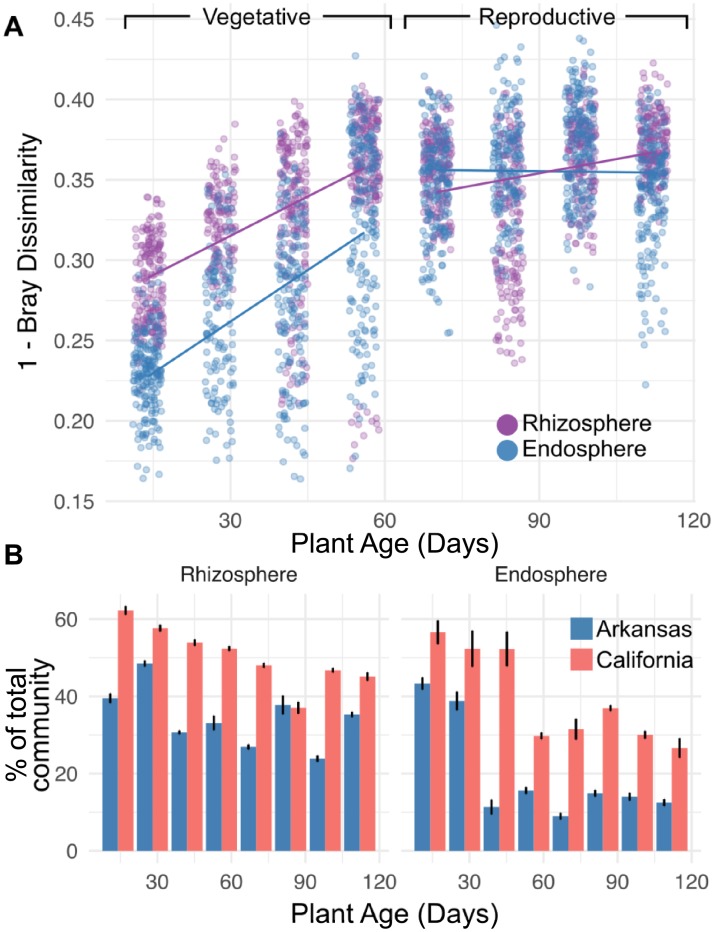
Rhizocompartments become more similar between field sites as a function of plant age. (A) Pairwise distances between each site within each common time point and each common compartment. The numerical values used to construct this plot can be found in S11 Data. (B) Mean total relative abundance of the site-specific operational taxonomic units (OTUs) within each time point. All OTUs found to be differentially abundant between sites within each common time point can be found in S12 Data. The numerical values used to construct panel B can be found in S13 Data.

Despite clear distinctions in microbiota composition between the growing regions, PERMANOVA indicated a significant interaction between plant age and the difference in microbiota composition between the sites (R2 = 0.019, *P* < 0.001, S1 Table). We found that both the endosphere and rhizosphere microbiota became significantly more similar over time between the 2 field sites (Fig 5A). Compared to the rhizosphere, the endosphere microbiota started the season as more dissimilar between the sites but reached comparable levels of similarity by the end of the season. This trend did not hold for the bulk soils (S5 Fig): the bulk soil communities between the sites did not show any change in similarity over time (*P* = 0.1), although we note that we were only able to sample bulk soil microbiomes over a 5-week span at the beginning of the season due to root proliferation later in the season. Interestingly, the similarity in microbial composition between the plants growing in each site stabilized after the rice plants had entered the reproductive phase. This result suggests that plants growing in disparate field sites initially acquire divergent root-associated microbiota, but the composition of these communities begins to converge throughout vegetative growth and maximizes and stabilizes during the reproductive phase. Together, these results suggest a host selection within the rhizosphere and endosphere that acts on similar microbes within the microbiota provided by the soil community.

Additionally, the plant age-predicting RF models were largely composed of OTUs that colonized roots later in the season at each field site. We hypothesized that this was due to the composition of the earlier time points being largely composed of site-specific OTUs. To test this hypothesis, we first identified OTUs in each time point whose abundance was skewed towards a specific site using edgeR, a software package that incorporates empirical Bayes methods to account for overdispersion common across count data [23]. After correcting for multiple comparisons, we identified 3,218 OTUs in the rhizosphere and 865 OTUs in the endosphere that were significantly skewed towards one of the field sites. We did not notice a clear trend in the number of OTUs called per time point (S6 Fig). When taking the overall relative abundance of the site-specific OTUs into account, however, we found that the site-specific OTUs made up a significantly greater proportion of the total microbiota of the earlier time points than the later time points in both field sites and in both the endosphere compartment and the rhizosphere compartment (Fig 5B, S6 Table). This effect was different across the rhizosphere and endosphere, with the endosphere having a more pronounced effect. This difference might be due to the rhizosphere hosting plant-responsive microbes as well as soil microbiota that do not respond to plant stimuli. Taken together, these results suggest that the initial colonization of the rhizosphere and endosphere is largely composed of site-specific OTUs; however, 8–9 weeks (56–63 days) after germination, a set of OTUs conserved between the 2 field sites begin to establish in the endosphere and rhizosphere, but not in the bulk soil.

### Developmental stage correlates with microbiota succession

**Fig 6:**
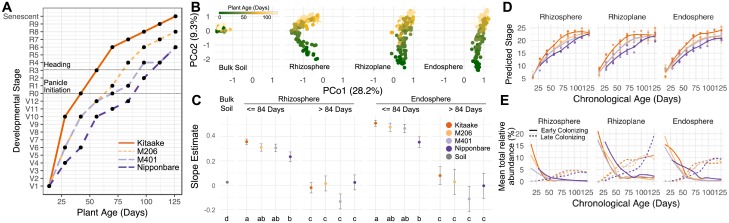
Varieties with different developmental rates have skewed microbiota progressions. (A) The developmental stage of the tested varieties as a function of plant age. We staged each variety using descriptors previously described [24]. R0 corresponds to the panicle initiation stage. It is important to note that M401 and M206 had nearly identical times to panicle initiation but afterwards diverged in time to heading. Data used to construct panel A can be found in S16 Data. (B) Principal coordinates analysis (PCoA) of the 2016 data indicating that root-associated compartment and plant age are major determinants of microbiota structure. The numerical values used to construct panel B can be found in S14 Data. (C) Linear slope estimates for the principal coordinate (PCo) 2 in panel B as a function of plant age for rhizosphere and endosphere compartments of each variety. Separate linear models were constructed for data prior to 84 days (corresponding to the sixth collection time point, the point at which all varieties had at least entered the panicle initiation stage) and for data after this time point. Letters under each point indicate statistical significance. Statistical tests were constrained to individual compartments and times of the season (prior to 84 days and after 84 days). The values used to construct panel C can be found in S15 Data. (D) The predicted developmental stage of the 2016 data as predicted by the stage-discriminant sparse random forest (RF) models. Developmental stage predictions used to construct panel D can be found in S16 Data. (E) Total relative abundance estimates for early and late colonizing stage-discriminant taxa between each cultivar and compartment. The values used to construct panel E can be found in S17 Data.

Our sparse RF models appeared to detect progressions in the root-associated microbiota that correlate with developmental transitions in the rice plants. However, plant developmental transitions also covary with climatic and edaphic factors, which may have indirect effects on the microbiota, making these factors difficult to uncouple from the direct effect of plant developmental stage on the root-associated microbiota. Because our California study was confined to 1 variety and our Arkansas study was confined to 2 varieties that have very similar developmental progressions, we were unable to unambiguously discriminate the effect of plant development from that of environmental factors on root-associated microbiota assembly. To investigate the direct effect of plant developmental stage on the root-associated microbiota, it is necessary to be able to distinguish the effect of plant age from developmental progression. With this aim, we grew 4 varieties from *O*. *sativa* sgp. *temperate japonica* at the same California field site during the 2016 season: Kitaake, California varieties M206 and M401, and Nipponbare. These 4 cultivars grow with different developmental progression rates (Fig 6A). The varieties were water seeded in the California field in a complete randomized block design. We sampled plants within each plot every 2 weeks throughout the season, collecting rhizosphere, rhizoplane, and endosphere fractions from the plant roots. We also scored the plants for their developmental stages. Our revised field design allowed us to also collect bulk soil samples throughout the entirety of the season, as compared to the 2014 and 2015 seasons that had restricted soil sampling due to the invasiveness of the rice roots. After sequencing the V4 region of the 16S rRNA gene and filtering out plastid OTUs, we obtained 11,986,615 total sequences comprising 10,547 OTUs from 469 samples.

The data from the California 2016 season had similar trends as exhibited in the 2014 and 2015 seasons in California as well as the field trial in Arkansas. We again used PERMANOVA to investigate which experimental factors explained a significant amount of variance in this dataset (S7 Table). The root-associated compartments hosted distinct microbiota (Fig 6B, R2 = 0.328, *P* < 0.001), and these microbiota varied significantly due to plant age (Fig 6B, R2 = 0.068, *P* < 0.001). We found that genotype had a very small overall effect on the root-associated microbiota (R2 = 0.010, *P* < 0.001). This level of variance is smaller than what we have previously detected in rice but is not surprising given that the included varieties were constrained to the *O*. *sativa* sgp. *temperate japonica* subgroup. We observed a significant statistical interaction between plant age and genotype (R2 = 0.008, *P* < 0.001), suggesting that the trends in microbiota shifts over the season differ depending on the genotype. This result was not observed in the Arkansas field trial, perhaps because the included varieties were very similar in their developmental progression rates. Moreover, we found that plant developmental stage explained more variance in our dataset than plant age (R2 = 0.082, *P* < 0.001), again suggesting that developmental stage is an important descriptor for root-associated microbiota assembly. To further inspect this observation, we asked whether there was a significant interaction between plant genotype and plant age along the second principal coordinate (PCo) of Fig 6B as a function of plant age. We used the second PCo because this axis best differentiated plant age. We hypothesized that if plant developmental rate were to have an effect on the root-associated microbiota, the faster-developing rice varieties would have greater slope estimates than the slower-developing varieties over the second PCo. For each compartment, we fit a linear model to the first 6 collection time points (corresponding to 84 days after germination, the point at which all varieties had entered the reproductive phase). For comparison, a second linear model was fitted to the remainder of the season. These were used to model progression along the second PCo axis as a function of plant age and genotype [15]. We compared models that contained an interaction term between plant genotype and age to models assuming identical slopes between the genotypes and found that for both the rhizosphere and endosphere, models with interaction terms significantly improved the accuracy of the early season models (*P* = 0.02, *P* = 0.005, respectively; S8 Table), though a significant improvement was not detectable for the rhizoplane (*P* = 0.51), possibly reflecting the lower consistency intrinsic to technical limitations of sampling this compartment [8]. However, the late season models did not exhibit similar improvements with inclusion of interaction terms (*P* > 0.05 for all compartments, S8 Table). These results suggest that the genotypes progress at different rates across the second PCo axis during the first 84 days of the season, but not for the remainder of the season.

When inspecting the slope estimates for each variety over the second PCo as a function of plant age for the first 84 days of the season, we observed that progression of the microbiota followed similar trends as the plant development rate variation between the cultivars (Fig 6C). Specifically, Kitaake and Nipponbare had significantly different slope estimates from each other in the rhizosphere and endosphere, while M401 and M206 had intermediate slope estimates that were not significantly different to the other varieties. Nevertheless, we did identify a significant difference between M206 and M401 microbiota, both prior to panicle initiation (R2 = 0.015, *P* = 0.006) and after panicle initiation (R2 = 0.013, *P* = 0.036), suggesting that genotypic differences in microbiota structure do not arise solely from differences in developmental progression. Additionally, there were no significant differences between the genotypes after 84 days on the second PCo axis, with each cultivar having a slope that was not significantly different than 0. Each compartment had a positive slope estimate over the first 84 days of the growing season, but unplanted soil had a lower slope estimate than the rhizocompartments. Taken together these results indicate that root microbiota shifts throughout the season appear to arise independently of shifts in the bulk soil, and the rate of these shifts in the rhizosphere and endosphere correlate with the developmental progression rate of the host plant.

We next used the sparse RF models generated from the California and Arkansas data to predict the age of samples taken from the 2016 season. In general, the models predicted the ages of the plants accurately (S7A Fig), indicating that root microbiota progressed in a similar manner in the California 2016 season as in the 2014 and 2015 seasons. The endosphere predictions showed significant variation due to cultivar (*P* = 0.001, ANOVA, S9 Table), while the rhizosphere predictions did not (*P* = 0.191, ANOVA, S9 Table). Furthermore, the endosphere model showed trends in the predictions that correlated with developmental progression rate variation between the cultivars with Nipponbare having predicted ages significantly younger than Kitaake and M206 (adjusted *P* = 0.0012 and *P* = 0.0021, respectively, S10 Table). When observing the total abundance of age-discriminant OTUs from the models between the cultivars over time, we found that late colonizing OTUs in the endosphere took a significantly longer period of time to establish in Nipponbare compared to Kitaake and M206 (adjusted *P* = 0.0002 and *P* = 0.0016, respectively; S7B Fig). These data suggest that the rhizosphere age-predicting model is largely robust to differences in phenology due to plant genotype but that the accuracy of the endosphere model is affected by variation in plant developmental rate.

To have a better understanding of which microbes are associated with the various developmental stages, we formed new RF models for predicting plant developmental stage rather than plant age. Development in plants is a continuous process marked by numerous stage-specific morphological features. We monitored the developmental stage of the genotypes throughout the season, assigning a numerical value from 1–27, with a value of 1 corresponding to a recently germinated seeding and 27 corresponding to a senescent plant (S11 Table). These values reflect a numerical value given to the stages used in Counce et al. to describe rice development [24]. Panicle initiation corresponded to a value of 18; thus, a plant that has entered the reproductive phase has a value of 18 or higher, and a plant in the vegetative phase has a value of 17 or lower. We followed the same approach as previously mentioned to develop the RF models. Briefly, we trained full RF models in each compartment where we regressed the full dataset of OTUs against the developmental stage number for a training set of samples. From these full models, we sequentially removed OTUs of lower importance while performing 10-fold cross validation. We found that the models were near peak accuracy when including 54 of the most important OTUs for each compartment. With these 54 most-important OTUs, we developed sparse RF models that model the microbiota as a function of plant developmental stage (Fig 6D). When the predicted values of plant developmental stage were plotted as a function of the plant’s chronological age, we found that the predictions accurately matched the developmental progressions that we witnessed in the field (see Fig 6A). Variation in developmental stage prediction slopes was significantly affected by plant genotype in each compartment (Rhizosphere *P* = 8.1 × 10^−8^, Rhizoplane *P* = 3.3 × 10^−5^, Endosphere *P* = 2.3 × 10^−2^). These results indicate that OTUs within the microbiota in each compartment can be used to predict plant developmental stage, even when developmental progression rates differ between rice cultivars.

We again classified the OTUs included in each stage-predicting RF model as early/late/complex colonizers using a linear model to detect whether the OTUs had significantly increasing, significantly decreasing, or complex distributions throughout the season. The phylogenetic classification of early/late colonizing OTUs mimicked those of the age-discriminant models (S2 and S8 Figs). Of the early colonizing OTUs over the season, 3 were shared between the compartment-specific models, while 9 of the late colonizing OTUs were shared. We next sought to address whether OTUs included in the models peak in abundance at different times in the season for the different cultivars. To do this, we calculated the average abundance of each OTU included in each model at each time point for each cultivar. For every compartment, we found that the early colonizing OTUs peaked later in Nipponbare than in the other cultivars. When taking the total abundance of increasing/decreasing OTUs into account, we found that the early colonizing OTUs tended to persist for a longer period of time in Nipponbare than in the other varieties (Fig 6E). Similarly, the OTUs classified as late colonizers took longer to establish in Nipponbare than in the other varieties. This pattern was consistent across each compartment. We note that the developmental stage sparse RF models used fewer OTUs than the plant age models to make accurate predictions. Together, these results indicate that both plant age and developmental stage are important drivers of the root microbiota.

## Discussion

Microbial communities associated with plants can have strong positive and negative effects on plant health and nutrition [4] as well as contribute to greenhouse gas emissions [25,26] and biogeochemical cycling [27]. Understanding and studying the spatial distribution as well as temporal progressions of the root microbiota is therefore an important aspect for crop and environmental improvement. Our analysis provides a detailed characterization of the spatiotemporal dynamics of the root microbiota and reveals insights into how plant development and abiotic stresses affect development on the microbiota.

### The dynamic patterns of root-associated microbiota are consistent across field sites and seasons

Previous studies conducted under greenhouse conditions have reported that compositions of plant microbiota are dynamic during plant growth [8,12,28,29]. Nevertheless, because of the limited extent of both temporal and spatial sampling in these studies, e.g., absence of data from the endosphere compartment [28,29], a comprehensive picture of the changes in the microbiome during the life cycle, as has been accomplished for humans, has yet to be formulated for plants. Additionally, the difference between greenhouse and field conditions is important given that nutrient dynamics and plant physiology vary under the 2 settings [30,31], and it has been demonstrated that plant microbiota are also altered by field conditions compared to greenhouse conditions [8]. Similarly, the importance of ecological factors driving community assembly is overestimated if the experimental observations are constrained to one soil type or season [32].

Here we demonstrate spatiotemporal shifts in microbiota composition during the life cycle that are consistent across multiple years of cultivation in the same field (Fig 1A–1C). Moreover, although we found large differences at the OTU level between the 2 tested field sites in California and Arkansas, at the phylum level we found that there were remarkable similarities in spatiotemporal profiles of microbiota abundance between the field sites despite large geographic distances, climatic differences, and cultivation practices (Fig 2A). In general, there were more phyla that were decreasing in relative abundance in the endosphere over the life cycle of the rice plants, while fewer phyla were increasing in relative abundance. In the rhizosphere, we found that more phyla were increasing rather than decreasing over the life cycle (Fig 2C). These data reinforce the exclusionary role of the endosphere compartment compared to the rhizosphere compartment as observed in other studies [6–8], but indicate that exclusion of microbes in the endosphere is age sensitive. *Betaproteobacteria* and *Deltaproteobacteria* were the dominant classes enriched in the root endosphere compared to the bulk soil. These 2 classes showed opposing patterns of abundance over the season: *Betaproteobacteria* was largely decreasing in abundance, while *Deltaproteobacteria* increased (Fig 2C).

Our high-resolution sampling scheme allowed us to deeply characterize the developmental patterns of microbiota over the growing season. The rhizoplane and endosphere microbiota progressed over the course of the first 7 to 8 weeks after germination but then stabilized in composition thereafter (Fig 1D). This was consistent across the 2 years of sampling from the California field as well as for samples collected from the Arkansas field.

### Creating a baseline for normal rice root microbiota progression

By employing a machine learning approach, we were able to model rice plant age as a function of fluctuating relative abundances of OTUs (Fig 3). Using the RF algorithm, we identified OTUs in the rhizosphere and endosphere compartments that were discriminant of plant age. Using these sets of OTUs, we were able to accurately predict the plant age of samples gathered from the California and Arkansas field sites. We were also able to use these models to accurately predict the age of rice plants grown in the California site in the 2015 season, despite the models not being trained on this data. These results indicate that groups of microbes proliferate predictably between field sites and between years. The sets of OTUs included in these models both increase and decrease in relative abundance over the course of the season (Fig 3B). The early and late colonizing OTUs were distinct at the phylum and order levels, suggesting that functional capabilities encoded by these microbes change throughout the season.

These results show that OTUs conserved between 2 diverse field sites can be used infer the age of rice plants. It is unknown whether our models developed for the rhizosphere and endosphere compartments are generalizable to rice plants grown in other regions of the world: climate, geography, and cultivation practices are all factors that contribute to microbiome structure and would likely affect the age-predicting models. The rice rhizosphere microbiota shows similarity at lower-resolution taxonomic levels [33], even when grown on different continents, so it is likely that RF models built at the order or family level may allow generalization in predictability across continental scales. Diverse plant species host divergent microbiota assemblages, even at the phylum level [34]; thus, it is unlikely that our models could be accurately applied to predicting the age of a set of genetically diverse plant species.

To test our hypothesis that environmental variables could affect the accuracy of age prediction, we used the age-predicting models to estimate the age of plants experiencing drought compared to those experiencing well-watered conditions (Fig 4). We found that the predicted age of drought-stressed plants using the endosphere models was significantly younger compared to well-watered plants, indicating that the water-deprived plants were hosting a developmentally immature endosphere microbiota (Fig 4A). This finding is intriguing given that rice plants experiencing drought stress during the vegetative stage typically have delayed flowering times [35–37]. These data support a model in which an arrest in host plant development caused by exposure to drought stress in turn impacts normal development of the endospheric microbiota. More studies will need to be conducted to confirm this hypothesis. Nevertheless, these results indicate that the models described here can be used to create a baseline for normal microbiota development and to test how environmental or biological perturbations may affect the maturation process. It is unknown how the OTUs included in each model relate to the overall health of the rice plants and assembly of a healthy microbiota. Age-discriminant microbes in the human gut have been found to be important for the health of the host [38–40]. In malnourished Bangladeshi children, the gut microbiota was shown to be underdeveloped compared to healthy children [38]. It was found that age-discriminant OTUs identified through RF models from the healthy human gut microbiota prevented growth defects in mice harboring microbiota from malnourished children [39]. It would be of interest to investigate whether isolated age-discriminant OTUs from plants could also play a role in recovery from biotic or abiotic stresses that delay developmental progression.

### Late colonizing OTUs show greater conservation between field sites

Over the course of the season, we showed that the rhizosphere and endosphere communities between field sites grow more similar, stabilizing around 8 to 9 weeks after germination (Fig 5A). Similarly, there were more age-discriminant OTUs in the rhizosphere and endosphere RF models that showed increasing trends in relative abundance over the course of the growing season (Fig 3B). These results suggested that there was less conservation between the field sites for early colonizing OTUs. Our data suggests that site-specific OTUs were significantly greater at the beginning of the season in both the rhizosphere and endosphere but were diminished at later time points in the growing season (Fig 5B). This effect was greater in the endosphere than in the rhizosphere, presumably because the rhizosphere is host to both microbes affected by plant processes as well as microbes from the soil biota that are unaffected by signals originating from the host. The increased conservation of the late-emerging microbiota may be due to plant selectivity, while the early colonizing microbiota may be due to opportunistic colonization of plant tissue by the soil microbiota. Nevertheless, through our RF approach, we were able to identify specific early colonizing microbes that are shared between the 2 field sites, and these OTUs were almost unanimously enriched in their respective rhizocompartments compared to bulk soil (S3 Fig), suggesting active or passive recruitment of the conserved early colonizing microbiota. In humans, the early colonizing gut microbiome has been implicated in educating the immune system [41,42]. In plants, it is unclear whether the conserved early colonizing microbiota plays a role in conditioning the activity of the plant innate immune system. A recent study in maize using a synthetic community found that certain bacteria, when omitted from the community, drastically disrupted normal microbiota assembly in roots and that disruption of the normal microbiota assembly led to greater susceptibility to fungal pathogens [43]. It may be possible that a portion of the conserved early colonizing taxa may be acting as such keystone species; thus, it is of interest to characterize isolated members from the early colonizing age-discriminant OTUs to understand whether they have a role in immune system function and microbiota assembly.

### Progression of the root-associated microbiota correlates with rice developmental progression

Previous studies have suggested that root microbiota are assembled early in the plant life cycle and are subsequently insensitive to the developmental status of the host plant [28,29]. Strong support for this model comes from the absence of significant differences in microbiota structure between an early flowering *A*. *alpina* mutant (*pep1*) and a nonflowering wild-type plant at the same age, implying that developmental stage is not responsible for microbiota compositional changes over time [12]. While this conclusion may hold across other perennial plant species, our results suggest that this conclusion might not be generalizable to all plants. By growing rice varieties with differing developmental trajectories, we were able to quantify the effect of plant development on the root-associated microbiota. We observed that there was a gradient in microbiota maturation across the second PCo in the rhizosphere and endosphere (Fig 6C). By using a RF regression, we were able to identity sets of microbes in each rhizocompartment that are able to distinguish the samples by developmental stage (Fig 6D). These results suggest that root microbiota are affected by both plant age and developmental stage and these effects influence different sets of microbes.

There are several likely factors that could cause the observed differences between our study and Dombrowski et al. *A*. *alpina* is a wild perennial plant, and rice is a domesticated annual species. One hallmark trait of cereal domestication is the selection for varieties with larger sink sizes in the seed [44]. For instance, in wild species, source carbohydrates are more evenly distributed to various sink tissues in the plant than in domesticated cereals, in which the seeds are a predominant sink [44,45]. The discrepancy between sink-source dynamics in these 2 host plants could at least partially explain why our study detected shifts in the microbiota due to developmental stage, while it was undetected in *A*. *alpina*. In rice, accumulation and storage of carbohydrates in the stems occur until the onset of reproduction, after which internal signals reprogram the host plant to repartition carbohydrates to the developing panicle and to the filling grain [46]. These host signals, along with the shifting nutritional needs at this stage, could explain the stabilization in the microbiota that occurs at the onset of reproduction.

### A model for acquisition and dynamics of the root-associated microbiota

Previous studies have found changes in the root-associated microbiota during the growth of the host plant [12,28,29,47], but the patterns of compositional changes have not been elucidated. The dense temporal and spatial sampling undertaken here reveals a clear pattern of root microbiome dynamics over the life cycle of an annual plant. The data, from multiple growing seasons and sites, are consistent with a 2-stage model that can be summarized as follows: immediately after germination, a community of early colonizing microbes becomes established inside and in the vicinity of the roots, representing a potential “juvenile microbiome.” It is unclear to what extent the host actively drives this process, as many of the early acquired microbes are site-specific and may represent opportunistic colonization by a subset of the soil microbiota. Nevertheless, a set of early colonizing microbes conserved between field sites were identified using our RF models and may represent consortia that are responsive to cues from the host plant. At around the time of entering the reproductive phase, a later colonizing set of microbes displaces the early colonizing microbes and then remains stable throughout the remainder of the life cycle of the host plant, potentially an “adult microbiome.” The rate of these transitions is dependent upon the rate of developmental progression, which in turn is genotype dependent. The microbes colonizing later in development were more conserved between the field sites, suggesting a greater influence of factors from the host. Root exudate composition varies significantly over the life cycle of Arabidopsis and tomato [48,49], and factors in exudates might underlie some of the shifts observed during the vegetative to reproductive transition. A more pronounced stabilization effect of the adult microbiome is observed in the rhizoplane and endosphere as compared to the rhizosphere (Fig 1D), raising the possibility of a more direct control by the host. The activity of the plant immune system can exhibit differences during plant growth, as demonstrated by the decreased susceptibility of some cereal crops to various pathogens during the adult phase [50–52]. Changes in the plant immune system are likely to play a larger role in affecting dynamics of the rhizoplane and endosphere microbiota than the rhizosphere, as microbes on the root surface and interior are in physical contact with the host plant cells.

In 1904, Lorenz Hiltner coined the term “rhizosphere” when he proposed that photosynthates exuded into the soil by plants likely alter the composition and abundance of microorganisms surrounding the roots [53]. Hiltner proposed that the communities of microorganisms in this zone impact plant nutrition and health [53,54] and that these communities may be affected by the stage of plant growth [55]. The role of rhizosphere microorganisms in plant nutrition and disease resistance has been confirmed [4,5]. Our results provide support for Hiltner’s hypothesis that the stage of plant growth is an important determinant of root-associated microbial composition.

Manipulation of the soil microbiota using bacterial isolates to increase crop yield and resistance to pathogens has been proposed as an enhancement to conventional plant breeding [56]. Using monoassociation assays, arrays of plant growth-promoting or disease-suppressing bacteria have been identified [57,58]. However, a major problem for translation to field conditions has been the inconsistent persistence of plant beneficial bacteria once inoculated into complex soil microbial communities [59–61]. Our findings that the composition of the root-associated microbiota are sensitive to plant age and developmental stages suggest that a good match with plant age might be a factor for persistence in the face of competing soil microbes. Thus, a plant growth-promoting bacterium that normally establishes during the reproductive phase might not be successful if supplied with seeds. The analysis provided here indicates that consideration of age appropriateness of a microbial inoculum could be utilized to enhance the efficacy of beneficial microbes for agricultural applications.

## Materials and methods

### Plant growth and sampling during the California 2014 and 2015 seasons

We collected samples from a commercially cultivated rice field in Arbuckle, California. In both seasons, the field was water seeded in early May. Water seeding is conducted by first preparing the field by removing winter vegetation, disking the soil to produce baseball-sized clods, applying nutrients, and then the field is flooded. The rice seeds are soaked in water overnight and then loaded into an aircraft, where they are then applied aerially to the field in an even density. For this particular field, the farmer grew the M206 cultivar, a medium-grained California variety that has an average heading date of 86 days after germination. We began sampling plants 7 days after the fields were seeded, which coincided with the emergence of the seminal roots. In the 2015 and 2016 seasons, we restricted our area of sampling to a 150×150 foot section of the field. Within this section, we sampled plants from random locations. Our sampling occurred as previously described [8]. Using gloved hands, we would scoop under the root mass to separate the plant from the ground. Grabbing the plant by the base of the stem, we would then shake the plant to remove loosely attached soil from the roots. We would then place the roots with tightly adhering soil into 50 mL Falcon tubes with 15 mL autoclaved phosphate buffered saline (PBS) solution. We then brought the samples back to the laboratory at UC Davis for subsequent processing.

### Plant growth during the Arkansas 2016 season

In the Arkansas 2016 season, we grew an *O*. *sativa* sgp. *tropical japonica* variety, Sabine, and a hybrid variety, CLXL745, in a split-plot design in a privately owned agricultural field near Jonesboro, Arkansas. Each plot was isolated from other plots via berms, and each plot had its own source of water and drainage (see design, S9 Fig). The roots of the plants were collected over the growing season as described above, placed into 50 mL Falcon tubes, and shipped overnight on ice to UC Davis. At UC Davis, the rhizosphere and endosphere compartments were separated (see below) and stored at −80 °C until DNA could be extracted. Due to the extra processing steps required to collect rhizoplane samples and the distance these root samples were shipped, we did not sequence the rhizoplane samples from the Arkansas field trial.

### Plant growth during the California 2016 season

We grew closely related *O*. *sativa* sgp. *temperate japonica* cultivars in the same field in Arbuckle, California, for the 2016 season in a Latin square design. Kitaake is a variety typically used under laboratory settings because of its relatively fast life cycle. M206 and M401 are varieties adapted to growing in California with medium times to panicle initiation. M401, however, requires a longer period of time for flowering. Nipponbare is a variety with a longer time to panicle initiation and flowering. Briefly, we designed a fully randomized block design to grow these varieties in 1×1 m plots in quadruplicate (see design, S10 Fig). We left 0.5-m-wide walking lanes between each plot, which subsequently allowed us to sample bulk soil throughout the course of the growing season. To be consistent with the previous years’ methods, we water seeded these varieties. This entailed soaking the seeds in a 2% bleach solution for 4 hours to remove the risk of the field being contaminated with *Fusarium moniliforme*, a seed-borne fungal pathogen that causes the disease Bakanae. The seeds were then washed 3 times with sterile water and soaked overnight. We then hand seeded each plot at a similar density as what the farmer had applied in previous seasons. At each time point, the plants were sampled as previously described and transported to the lab for further processing. The plants during this season were dissected to ascribe various developmental stages in accordance with Counce et al. [24]. Because the rice plants were water seeded, there was a high chance that each genotype’s plot could be contaminated by seeds drifting in from another plot or seeds that the field manager had planted. The genotypes were confirmed by amplifying SSR marker RM144 [62] using the endosphere DNA samples collected from the plants. From this analysis, we excluded the first week of Nipponbare samples from the analysis because of all of the samples being contaminated by M206. Similarly, we removed other samples from the analysis in which the SSR marker genotyping did not match the genotype of the plot in the field. All soil chemical analyses were conducted at the University of California, Davis Analytical Laboratory (S2 Table). Soil classifications were obtained from the US Department of Agriculture web soil survey (https://websoilsurvey.sc.egov.usda.gov/App/HomePage.htm).

### Separation of the root-associated compartments and DNA extraction

In each instance of sampling roots, we collected material from the first inch of roots just below the root-shoot junction. The root-associated compartments were separated as previously described [8]. The roots with soil attached were vortexed in PBS solution, and 500 uL of the resulting slurry was used for DNA extraction. The roots were cyclically washed in fresh PBS solution until no soil particles were visible in the solution. The roots were then placed into fresh PBS and sonicated for 30 seconds to remove the surface cell layer of the roots. The resulting slurry was centrifuged down to concentrate the biomass and then used as the rhizoplane fraction for DNA extraction. The remaining roots were sonicated twice more, refreshing the PBS solution at each stage, and then ground in a bead beater. The resulting solution was used for DNA extraction as the endosphere fraction. All DNA extraction was performed using the MoBio Powersoil DNA isolation kits.

### PCR amplification and sequencing

We amplified the V4 region of the 16S ribosomal RNA gene using the universal 515F and 806R PCR primers. Both our forward and reverse primers contained 12 base-pair barcodes, thus allowing us to multiplex our sequencing libraries at over 150 libraries per sequencing run [8]. Each library was accompanied by a negative PCR control to ensure that the reagents were free of contaminant DNA. We purified the PCR products using AMPure beads to remove unused PCR reagents and resulting primer dimers. After purification, we quantified the concentration of our libraries using a Qubit machine. Our libraries were then pooled into equal concentrations into a single library and concentrated using AMPure beads. The pooled library then went through a final gel purification to remove any remaining unwanted PCR products. Pooled libraries were sequenced using the Illumina MiSeq machine with 250×250 paired-end chemistry.

### Sequence processing, OTU clustering, and OTU filtering

The resulting sequences were demultiplexed using the barcode sequences by in house Python scripts. For the case of the drought data, we downloaded the sequences for the Short Read Archive project number PRJNA386367. The sequences were quality filtered and then assembled into full contigs using the PandaSeq software [63]. Any sequences containing ambiguous bases or having a length of over 275 were discarded from the analysis. The high-quality sequences were clustered into OTUs using the Ninja-OPS pipeline [64] against a “concatesome” composed of the Greengenes 97% OTU representative sequence database (version 13_8) [65] and then assembled into an OTU table. This OTU table was filtered to remove plastidial and mitochondrial OTUs. To account for sequencing depth differences between the samples, each OTU in each sample was divided by the total sequencing depth for the respective sample and multiplied by 1,000, resulting in a relative abundance in units of per mille. OTUs that occurred in less than 5% of the samples were filtered from the table (S11 Fig). This process reduced the total OTU count from 24,048 to 10,893 taxa. The resulting 10,893 taxa were used for the analysis.

### Statistical analysis

All statistical analyses were conducted using R version 3.1 [66]. Unless otherwise noted, we determined statistical significance at ɑ = 0.05 and, where appropriate, corrected for multiple hypothesis testing using the Bonferroni method. Shannon diversity was calculated using the diversity() function, unconstrained PCoA analyses were conducted using the Vegan capscale() function by specifying an intercept-only model (R Code: capscale(log2(RA) ~ 1), and PERMANOVA was conducted using the adonis() function from the Vegan package [67]. Linear models were run using the lm() function, and ANOVA was run using the aov() function from the Stats package [66]. Beta regression was performed using the BetaReg package [68]. RF models were generated and analyzed using the randomForest package [69]. Differential OTU abundance was performed using exact tests in the package edgeR [23]. All graphs and plots were generated using the ggplot2 package [70]. R notebooks for the full analyses can be found at https://github.com/bulksoil/LifeCycleManuscript.

### Generation of sparse RF models

To model plant age as a function of microbiota composition, we began by developing compartment-specific full RF models for both the endosphere and rhizosphere samples by regressing the relative abundance of all OTUs against the age of the plants from which the samples were collected. For our training data, we selected samples from the California 2014 and Arkansas 2016 data. Half of the samples from each time point and rhizosphere and endosphere compartments were randomly sampled for the training set. From these full models, we were able to rank individual OTUs by their importance in contributing to the accuracy of age prediction by the models. This process is performed by permuting the relative abundance levels for an OTU and calculating the increase in mean squared error of the model. OTUs whose relative abundances when permuted yield increased errors in the model are considered to be important to the accuracy of the model. This step was performed using the importance() command from the randomForest R package. Because not every OTU included in the full RF models will contribute to the accuracy of the models, we next performed 10-fold cross validation while simultaneously removing less important OTUs to evaluate model performance as a function of inclusion of the top age-discriminant OTUs using the rfcv() function in the randomForest R package. We found that there was a minimal increase in accuracy when including more than 85 of the most important OTUs (S12 Fig). The top 85 OTUs from the full RF model of each compartment were then used as inputs for sparse RF models for each compartment with no further parameterization.

### Data deposition

All raw sequences derived from this project were submitted into the Short Read Archive of NCBI and can be found under the BioProject accession number PRJNA392701. OTU tables, metadata files, taxonomy files, and R data files are available from the Dryad Digital Repository: https://doi.org/10.5061/dryad.7q7k1 [71].

## Supporting information

S1 Data Numerical values for Fig 1A. (XLSX)

S2 Data Numerical values for Fig 2A. (XLSX)

S3 Data Beta regression coefficients for Fig 2B and 2C. (XLSX)

S4 Data Numerical values for Fig 3A. (XLSX)

S5 Data Slope estimates for operational taxonomic unit (OTU) abundance classification in Fig 3B. (XLSX)

S6 Data Taxonomic classification for operational taxonomic units (OTUs) in S3 Fig. (XLSX)

S7 Data Numerical values for Fig 3C. (XLSX)

S8 Data Numerical values for Fig 4A. (XLSX)

S9 Data Tukey’s honest significance test results for the drought experiment comparisons. (XLSX)

S10 Data Numerical values for Fig 4B. (XLSX)

S11 Data Numerical values for Fig 5A. (XLSX)

S12 Data List of differentially abundant operational taxonomic units (OTUs) between sites used to construct Fig 5B. (XLSX)

S13 Data Numerical values for Fig 5B. (XLSX)

S14 Data Numerical values for Fig 6B. (XLSX)

S15 Data Numerical values for Fig 6C. (XLSX)

S16 Data Numerical values for Fig 6A. (XLSX)

S17 Data Numerical values for Fig 6E. (XLSX)

S18 Data Numerical values for S2 Fig. (XLSX)

S19 Data Numerical values for S4 Fig. (XLSX)

S20 Data Numerical values for S5 Fig. (XLSX)

S21 Data Numerical values for S7A Fig. (XLSX)

S22 Data Numerical values for S7B Fig. (XLSX)

S23 Data Numerical values for S8 Fig. (XLSX)

S24 Data Numerical values for S11 Fig. (XLSX)

S25 Data Numerical values for S12 Fig. (XLSX)

S1 Fig Stabilization in microbiome dynamics occurs in multiple sites and seasons. (A) Heatmap of pairwise similarities between time points for the California field site in the 2015 season. (B) Heatmap of pairwise similarities between time points for the Arkansas field site in 2016.
(EPS)

S2 Fig Alpha diversity metrics across compartments, sites, and seasons. Data used to construct this figure can be found in S18 Data.
(PDF)

S3 Fig Decreasing, complex, and increasing age-discriminant operational taxonomic units (OTUs) are divergent at the order level. Full taxonomies for the age-discriminant OTUs can be found in S6 Data.
(EPS)

S4 Fig Age-discriminant operational taxonomic units (OTUs) with decreasing abundance over time are enriched in their respective rhizocompartments compared to bulk soil. Data used to construct this figure can be found in S19 Data.
(EPS)

S5 Fig Bulk soil communities do not become more similar between field sites over time. Pairwise Bray dissimilarity measures between bulk soil samples within the same age across the Arkansas 2016 and California 2014 field sites. Data used to construct this plot can be found in S20 Data.
(EPS)

S6 Fig Number of differentially abundant operational taxonomic units (OTUs) between the Arkansas and California field sites over time. Data used to construct this plot can be found in S12 Data.
(EPS)

S7 Fig California 2016 samples predicted by the sparse age-discriminant random forest (RF) models. (A) Age predictions for the rhizosphere and endosphere using the sparse RF models. Data used to construct this panel can be found in S21 Data. (B) Relative abundance of increasing/complex/decreasing age-discriminant operational taxonomic units (OTUs). Data used to construct this panel can be found in S22 Data.
(EPS)

S8 Fig Phylum and order distribution of the developmental stage discriminant random forest models. Data used to construct this figure can be found in S23 Data.
(EPS)

S9 Fig Layout for the Arkansas 2016 field study. Note that the map is not drawn to scale. The individual plots for each variety were approximately 20×20 ft.
(EPS)

S10 Fig Layout for the California 2016 field study. (EPS)

S11 Fig Distribution of average operational taxonomic unit (OTU) relative abundance as a function of prevalence. OTUs that were not observed in at least 5% of the samples were discarded and colored in red. OTUs that were observed in 5% or greater of the samples were kept for analysis and are colored black. Data used to make this plot can be found in S24 Data.
(TIFF)

S12 Fig Ten-fold cross validation errors for rhizosphere and endosphere random forest models as a function of inclusion of operational taxonomic units (OTUs) based upon their importance for model accuracy. A dashed vertical line has been drawn on the *x*-axis at the point of including 85 of the most important age-discriminant OTUs. Data used to construct this plot can be found in S25 Data.
(EPS)

S1 Table Permutational multivariate analysis of variance (PERMANOVA) results for California 2015–2015 and Arkansas 2016 data. (XLSX)

S2 Table Chemical content of tested soils. (XLSX)

S3 Table Order of operational taxonomic units (OTUs) plotted in Fig 3B. (XLSX)

S4 Table ANOVA results from testing the effects of drought on predicted microbiota age from drought and well-watered rice plants. (XLSX)

S5 Table Post hoc Tukey's honest significant difference test of differences between abundances of classifications of age-discriminant operational taxonomic units (OTUs) from drought and well-watered plants. (XLSX)

S6 Table Linear model results for abundance of site-specific taxa over the season in California and Arkansas. (XLSX)

S7 Table Permutational multivariate analysis of variance (PERMANOVA) results for California 2016 data. (XLSX)

S8 Table ANOVA results for the interaction effect of genotype on age progression along MDS2 of the principal coordinates analysis (PCoA) in Fig 6B. (XLSX)

S9 Table ANOVA results of age predictions of the samples for California 2016. (XLSX)

S10 Table Post hoc Tukey's honest significant difference tests between the predicted ages of cultivars using random forest model in the endosphere. (XLSX)

S11 Table Conversion table for discrete developmental stages to numerical values. (XLSX)

## Abbreviations

OTU: operational taxonomic unit
PCo: principal coordinate
PCoA: principal coordinates analysis
PERMANOVA: permutational multivariate analysis of variance
RF: random forest
sgp: subgroup
