# Supplementary figures and images for: Compositional shifts in root-associated bacterial and archaeal microbiota track the plant life cycle in field-grown rice
Source: PLoS Biol. 2018 Feb 23;16(2):e2003862. doi: 10.1371/journal.pbio.2003862 (PMC5841827; doi:10.1371/journal.pbio.2003862)

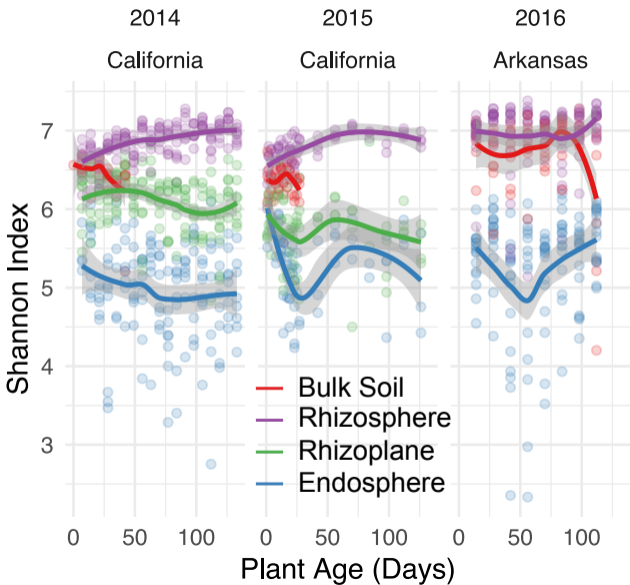

Supplement: S2 Fig — Data used to construct this figure can be found in S18 Data. (PDF) [file pbio.2003862.s027.pdf]

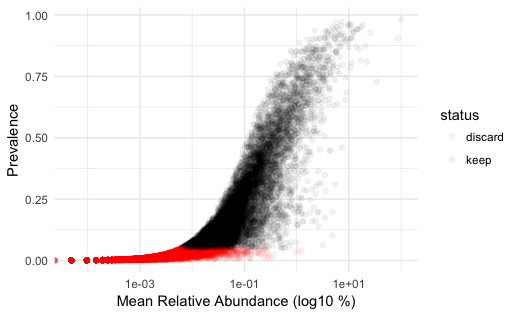

Supplement: S11 Fig — OTUs that were not observed in at least 5% of the samples were discarded and colored in red. OTUs that were observed in 5% or greater of the samples were kept for analysis and are colored black. Data used to make this plot can be found in S24 Data. (TIFF) [file pbio.2003862.s036.tiff]
